# STING Driving Synaptic Phagocytosis of Hippocampal Microglia/Macrophages Contributes to Cognitive Impairment in Sepsis‐Associated Encephalopathy in Mice

**DOI:** 10.1111/cns.70166

**Published:** 2024-12-19

**Authors:** Xin Lv, Min Jia, Xiao Feng, Jia‐xiong Jian, Jian‐jun Yang, Da‐qing Ma, Mu‐huo Ji, Yu‐gang Diao, Jin‐chun Shen

**Affiliations:** ^1^ Department of Anesthesiology, Pain and Perioperative Medicine The First Affiliated Hospital of Zhengzhou University Zhengzhou China; ^2^ Department of Anesthesiology, Jinling Hospital, Affiliated Hospital of Medical School Nanjing University Nanjing China; ^3^ Division of Anaesthetics, Pain Medicine & Intensive Care, Department of Surgery & Cancer, Faculty of Medicine Imperial College London, Chelsea & Westminster Hospital London UK; ^4^ Perioperative and Systems Medicine Laboratory Children's Hospital, Zhejiang University School of Medicine, National Clinical Research Center for Child Health Hangzhou China; ^5^ Department of Anesthesiology The Second Affiliated Hospital of Nanjing Medical University Nanjing China; ^6^ Department of Anesthesiology General Hospital of Northern Theater Command Shenyang China

**Keywords:** C1q, microglia/macrophages, sepsis‐associated encephalopathy, stimulator of interferon genes, synaptic plasticity

## Abstract

**Background:**

Sepsis‐associated encephalopathy (SAE) is a serious neurologic complication in septic patients with poor prognoses. There is increasing evidence that stimulator of interferon genes (STING) plays a crucial role in neuroinflammation and cognitive impairment. However, whether sepsis associated with STING changes contributes to cognitive impairment is unknown.

**Methods:**

Male adult mice received lipopolysaccharide (LPS) injection (a single dose of 4 mg/kg; i.p. injection) and 30 min later, they were injected with STING inhibitor C‐176 (a single dose of 30 mg/kg, i.p. injection). Behavioral assessments, biochemical measurements, in vivo and ex vivo electrophysiology techniques were conducted to investigate the association between LPS‐induced STING overexpression and cognitive function.

**Results:**

Cognitive impairment was associated with STING overexpression and activation of microglia/macrophages. Phagocytosis of microglia/macrophages as well as complement C1q release were increased after LPS injection, leading to abnormal pruning synapses, synaptic transmission reduction, long‐term potentiation (LTP) impairment, as well as abnormal theta oscillation in the hippocampus. Notably, STING inhibitor C‐176 significantly reversed these changes.

**Conclusions:**

Sepsis‐induced STING overexpression in microglia/macrophages may lead to synaptic loss, abnormal theta oscillation and LTP impairment through microglia/macrophages activation and complement C1q modulation, ultimately resulting in cognitive impairment.

## Graphical abstract

Injection of LPS led to hippocampal CA1 STING overexpression and microglial activation, which upregulated complement C1q‐mediated synaptic phagocytosis, resulting in abnormal theta oscillation and decreased LTP, ultimately leading to cognitive impairment.

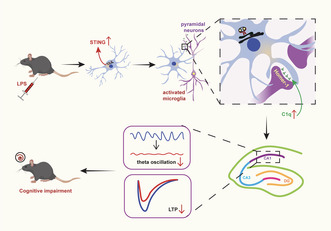

## 1 Introduction

Sepsis‐associated encephalopathy (SAE) is a diffuse brain dysfunction without direct central nervous system (CNS) infection [1]. Approximately 70% of patients with bacteremia developed neurologic symptoms ranging from lethargy to coma, and over 80% had abnormalities of electroencephalogram (EEG) [2]. The pathophysiology includes various factors such as endothelial injury, inflammation, cerebral ischemia, blood–brain barrier (BBB) injury, abnormal synaptic function, and excitatory toxicity [3]; yet the exact mechanism is not very clear. Therefore, the underlying pathogenesis needs to be investigated to develop an effective treatment to reduce the incidence of SAE.

Stimulator of interferon genes (STING) encoded by transmembrane protein 173 is an endoplasmic reticulum transmembrane protein [4]. DNA‐activated cyclic GMP‐AMP synthase (cGAS) uses adenosine triphosphate and guanosine triphosphate as substrates to generate the second messenger cyclic GMP‐AMP (cGAMP), which binds and activates STING [5]. Subsequently, activated STING recruits the phosphorylates of TANK‐binding kinase 1, which further activates interferon regulatory factor 3, ultimately upregulating the expression of type I interferon and enhancing inflammatory response through recruiting and activating immune cells [6]. The STING signaling pathway has been reported to play a role in the innate immune response, antitumor immunity, and inflammatory diseases [7, 8]. Microglia/macrophages in the brain can promptly transition to be an activated state from a rest state upon injurious stimulation. Subsequently, they undergo rapid proliferation, migration, phagocytosis, and cytokine secretion. It has been reported that the activation of microglia/macrophages was closely associated with cognitive impairment in diseases and disease conditions of the CNS [1]. Previous studies have demonstrated that the activation of STING triggered the transcriptional state of reactive microglia and promoted neurodegeneration, and cognitive impairment [4]. An increased level of STING were predominantly detected in microglia/macrophages within the damaged cortex following LPS administration, and similar cellular localization patterns were also reported in cerebral ischemia/reperfusion [9] and subarachnoid hemorrhage models [10]. Nevertheless, it remains unclear whether STING activation in microglia/macrophages contributes to the occurrence of SAE.

Synapses are the key sites of functional connections and information transfer between neurons, and synaptic plasticity is the cellular and morphological basis of learning and memory [11]. Accumulating evidence demonstrates that glial cells, in particular microglia/macrophages, play an important role in the formation and maintenance of neuronal circuitry including synapse elimination [12, 13]. Normal microglia/macrophages responses in brain tissue are necessary for phagocytosis to remove damaged neuronal cells and tissue debris, but a sustained or violent microglia/macrophages responses are detrimental [14]. Complement C1q, a member of the immune complement system, plays a central role in the selective pruning of synapses under phagocytosis by microglia/macrophages [15, 16]. Therefore, this study aimed to investigate whether microglia/macrophages activation by STING overexpression in the hippocampus leads to an increase of C1q secretion and excessive phagocytosis of synapses and ultimately causing cognitive impairment in an SAE model of mice.

## 2 Materials and Methods

### 2.1 Animals

Male C57BL/6J mice (10–12 weeks old and weighing 25–30 g) were obtained from Jiangsu Huachuang Sino Biotechnology Company, Nanjing, China. Five mice per cage were housed in an environment with a temperature of 21°C ± 3°C and humidity of 50% ± 10% with a 12 h light/dark cycle (light on 07:00 a.m.–07:00 p.m.). Access to water and food was ad libitum. All animal procedures were approved by the Animal Care and Use Committee of Jinling Hospital, Affiliated Hospital of Medical School, Nanjing University, Nanjing, China (No. 2023QJQNPY001).

### 2.2 Animal Model of SAE

Mouse was intraperitoneally injected with either lipopolysaccharide (LPS; 4 mg/kg) (the LPS group) or 0.9% sterile saline (the control group) [17]. LPS, diluted to be 0.4 mg/mL with sterile saline, was injected at an injection volume of 0.25–0.30 mL. If died or had no sign of sickness after LPS injection, they were excluded for further tissue and data analysis.

### 2.3 Experimental Designs and Drug Treatment

This study was completed in two separate experiments. In experiment 1, mice were randomly divided into the Con group (*n* = 12) and the LPS group (*n* = 12). In experiment 2, 140 mice were randomly divided into four groups: Con + Vehicle group (Saline + Vehicle), Con + C‐176 group (Saline + C‐176), LPS + Vehicle group (LPS + Vehicle), and LPS + C‐176 group (LPS + C‐176); each group consisted of 35 mice. STING inhibitor C‐176 (30 mg/kg, MedChemExpress, USA) or Vehicle (1% DMSO + corn oil) were administered intraperitoneally at 30 min after LPS or sterile saline injection [18, 19].

### 2.4 Open Field Test (OFT)

The motor ability of mice was measured by OFT. A white open field analysis box (40 cm × 40 cm × 40 cm) was used for OFT. The bottom surface was divided into 16 squares of equal area; the squares along the wall of the box were called peripheral grids, and the rest were central grids. Mice were placed in the central cell and allowed to explore freely for 10 min.

### 2.5 Y Maze Test

The working memory ability of mice was measured by Y maze test which consists of three arms, A, B, and C, at an angle of 120 to each other. Mice were placed from the center and allowed to explore freely for 8 min, and the order and total number of times they entered each arm were recorded. When the mice entered three arms in sequence, such as ABC, BCA, and CBA, it was defined as the correct spontaneous alternation. The correct rate of spontaneous alternation was calculated according to the following formula: Alternation (%) = number of correct spontaneous alternation entry arms/(total number of entry arms − 2) × 100%.

### 2.6 Novel Object Recognition (NOR) Test

The learning memory ability of mice was measured by NOR test, including habituation, learning, and test phases, which was conducted for 3 days. In the habituation phase, mice were put into the open box for adaptation without any objects for 10 min. In the learning phase, mice were placed in the box with their backs to the objects, and the exploration of the two identical objects of mice within 10 min was recorded. In the test phase, one of the objects was replaced with a new object with a completely different shape and color, and the exploration of the old and new objects within 10 min was recorded. The discrimination index is calculated as DI = *N*/(*N* + *F*) × 100%. (*N*: time spent exploring new object of mice; *F*: time spent exploring old object of mice).

### 2.7 Western Blot Analysis

Mice were deeply anesthetized with 3% isoflurane and killed via decapitation. Both hippocampal tissues of mice were harvested and then added with precooled protein lysate, and the protein concentration was determined by the BCA method. Sample 10 μg per well was used for electrophoresis transfer and the film was incubated with 5% skim milk for 1 h and washed with TBST three times, and then incubated with one of the primary antibodies: STING (ab288157, Abcam), ionized calcium‐binding adaptor molecule 1 (Iba1) (10904‐1‐AP, Proteintech), homer scaffolding protein 1 (Homer‐1) (160003, Synaptic Systems), C1q (11602‐1‐AP, Proteintech), GAPDH (60004‐1‐1 g, Proteintech), and Tubulin (A18C9‐1, Beyotime) overnight in a shaker at 4°C. After three rinses of TBST, secondary antibody was incubated at room temperature for 1 h and rinsed three times with TBST. This is followed by ECL color development, machine development, and image acquisition. The band intensities were determined with ImageJ software, and its expression content was reflected by the ratio of target protein relative to Tubulin or GAPDH.

### 2.8 Immunofluorescence

Under 3% isoflurane anesthesia, mice were injected with 40 mL phosphate buffered saline (PBS) followed by 40 mL 4% paraformaldehyde (PFA) through the left ventricle. Their whole brains were fixed in 4% PFA for 6–8 h and then transferred to 30% sucrose for overnight dehydration. The tissues were then embedded with OCT and frozen at −80°C. Coronal sections (thickness 30 μm) were made using a frozen microtome (−20°C). The slices were transferred to 24‐well plates, washed with PBS for three times, and then incubated with 10% goat serum containing 0.3% TritonX‐100 at room temperature for 1 h. Subsequently, antibodies were added: Iba1 (234 009, SYSY), neuronal nuclei (NeuN) (834502, BioLegend), glial fibrillary acidic protein (GFAP) (ab4674, Abcam), STING (ab288157, Abcam), Homer‐1 (160 003, Synaptic Systems), CD68 (137001, BioLegend), C1q (ab255973, Abcam), transmembrane protein 119 (TMEM119) (98778S, Cell Signaling), and incubated overnight in a shaking bed at 4°C. After washing with PBS for three times, the membrane was incubated with secondary antibodies at room temperature for 2 h, rinsed three times with PBS, and sealed with DAPI (1:1000; Beyotime Biotechnology) tablets. Micrographs were made with Zeiss LSM880 confocal microscope and ImageJ software was used for data analysis.

### 2.9 Golgi Staining

FD Rapid Golgi staining kit (PK401, FD NeuroTechnologies Inc. Company) was used for Golgi staining [20]. Mice were killed by decapitation under 3% isoflurane anesthesia and their brains were harvested immediately. Coronal slices with a thickness of 110 μm were made. Images were taken by optical microscope and analyzed by ImageJ software. Five mice were randomly selected from each group. For analysis, two hippocampal cornu ammonis 1 (CA1) pyramidal neurons and three spines were randomly chosen from each animal. Hippocampal CA1 neuronal morphology was analyzed with plugins NeuronJ and sholl analyses in ImageJ software. Dendritic spines were counted using the Cell Counter plugin in ImageJ software.

### 2.10 In Vivo Electrophysiology

The mice were anesthetized with 1.25% tribromoethanol (0.3 mL/10 g) and placed on a stereotactic instrument (RWD instrument). Anterior coordinates, posterior coordinates, and mesolateral coordinates were measured from the fontanel, while depth was calculated relative to the brain surface. Cranial nails were placed on one side of the skull, a 2.5 × 2.5 mm bone window was made on the other side of the skull surface, and the dural membrane was carefully torn and exfoliated. Eight‐channel microelectrode (Kd‐micro‐008, KedouBC) was implanted into the hippocampal CA1 (anteroposterior: −2.2 mm, mediolateral: −1.5 mm, and dorsoventral: −1.5 mm). Polysporin (Pfizer, Canada) was applied to exposed scalp wounds after surgical procedures for pain relief and to prevent infection. In our study, the EEG bands were classified as delta oscillations (0.5–3 Hz), theta oscillations (4–7 Hz), alpha oscillations (8–13 Hz), and beta oscillations (14–29 Hz). These signals were filtered by a passband of 0.3–300 Hz and further amplified and digitized at 2 kHz. All data analysis was performed using NeuroExplorer (Version 5, Plexon Corporation, Dallas, TX) software.

### 2.11 In Vitro Electrophysiology

Mice were first anesthetized using 5% isoflurane. Following this, their entire brain was carefully extracted and immersed in sucrose‐rich artificial cerebrospinal fluid (ACSF) maintained at a temperature of 0°C. The ACSF contained the following components: 126 mM NaCl, 2.5 mM KCl, 1 mM MgCl_2_, 1 mM CaCl_2_, 1.25 mM KH_2_PO_4_, 26 mM NaHCO_3_, and 20 mM glucose. Next, the brain was coronally sectioned according to the specifications outlined in the brain atlas. The cutting procedure was performed in sucrose‐rich ACSF with oxygenation conditions comprising 95% O_2_ and 5% CO_2_. Each brain slice had a thickness of 250 μm, and the cutting speed was set at 0.14 mm/s. These hippocampal slices were then transferred to ACSF, ensuring adequate oxygenation, and incubated at room temperature for a minimum of 45 min to ensure complete recovery of neuronal activity. For further examination, the hippocampal slices were placed on a Nikon orthomosaic microscope equipped with a stage containing a 10 × 40 water immersion objective. To stimulate the tissue, an electrode was positioned in the schafer collateral (SC), delivering both test and conditioned stimulation. Meanwhile, a recording electrode was placed 200–300 μm away from the stimulation electrode, specifically in the stratum radiatum of CA1. The baseline was set at 40%–50% of the maximum excitatory postsynaptic potential (EPSP) value. Following a stabilization period of 10 min, long‐term potentiation (LTP) was induced using three sets of high‐frequency stimulation (HFS) at 50 Hz with 100 pulses. The potential slope was then normalized against the mean baseline value. A SutterPatch‐IPA2 amplifier was utilized for data recording, and signals ranging from 0 to 10 kHz were selected while being filtered through a 5 kHz low‐pass filter. Subsequently, Igor Pro9.0 software was employed for data analysis.

To record miniature excitatory postsynaptic currents (mEPSCs), the whole‐cell patch‐clamp technique was applied. Brain slices from mice were placed in a recording chamber and perfused with artificial cerebrospinal fluid that had been previously bubbled with 95% O_2_ and 5% CO_2_. Whole‐cell recordings were conducted on pyramidal neurons within CA1, which were visualized using an upright microscope equipped with a × 40 water‐immersion lens (Olympus BX51W1, Japan) and an infrared‐sensitive CCD camera. The patch pipette, with an input resistance of 3–6 MΩ, contained a solution consisting of 125 mM potassium D‐gluconate, 8 mM NaCl, 0.2 mM EGTA, 10 mM HEPES, 2 mM Mg‐ATP, and 0.3 mM Na‐GTP. Upon break‐in, series resistances typically ranged from 15 to 30 MΩ and were compensated by 70%. Pyramidal neurons with stable series resistance (showing no more than 20% changes throughout the recording) were chosen for further analysis. An Axon patch 700B amplifier was used for data collection, with low‐pass filtering set at 2 kHz. The data were digitally sampled at 10 kHz and later analyzed using Clampfit software (Molecular Devices). To evaluate the intrinsic membrane properties of neurons, their spiking patterns were recorded in the current‐clamp configuration. This was achieved by injecting a series of current pulses with a duration of 400 ms and an intensity ranging from 60 to 240 pA, incremented by 60 pA. To isolate mEPSCs, the bath solution was supplemented with tetrodotoxin (1 μM) and bicuculline (10 μM). The Mini Analysis Program (Version 6.0.3, Synaptosoft) was utilized to analyze the mEPSCs, with all events detected above a threshold of 5 pA.

### 2.12 Statistical Analysis

Statistical tests were performed with GraphPad Prism 9.0 software. Data distribution was assessed with the Shapiro–Wilk test and they were then presented as mean ± standard deviation (SD) [21] together with dot plot and then analyzed using two‐tailed Student's *t*‐test or two‐way ANOVA with Tukey's post hoc test as appropriate. A value of *p* < 0.05 was considered to be statistically significant.

## 3 Results

### 3.1 STING Signaling Was Predominantly Activated in Microglia/Macrophages in SAE Mice

**FIGURE 1:**
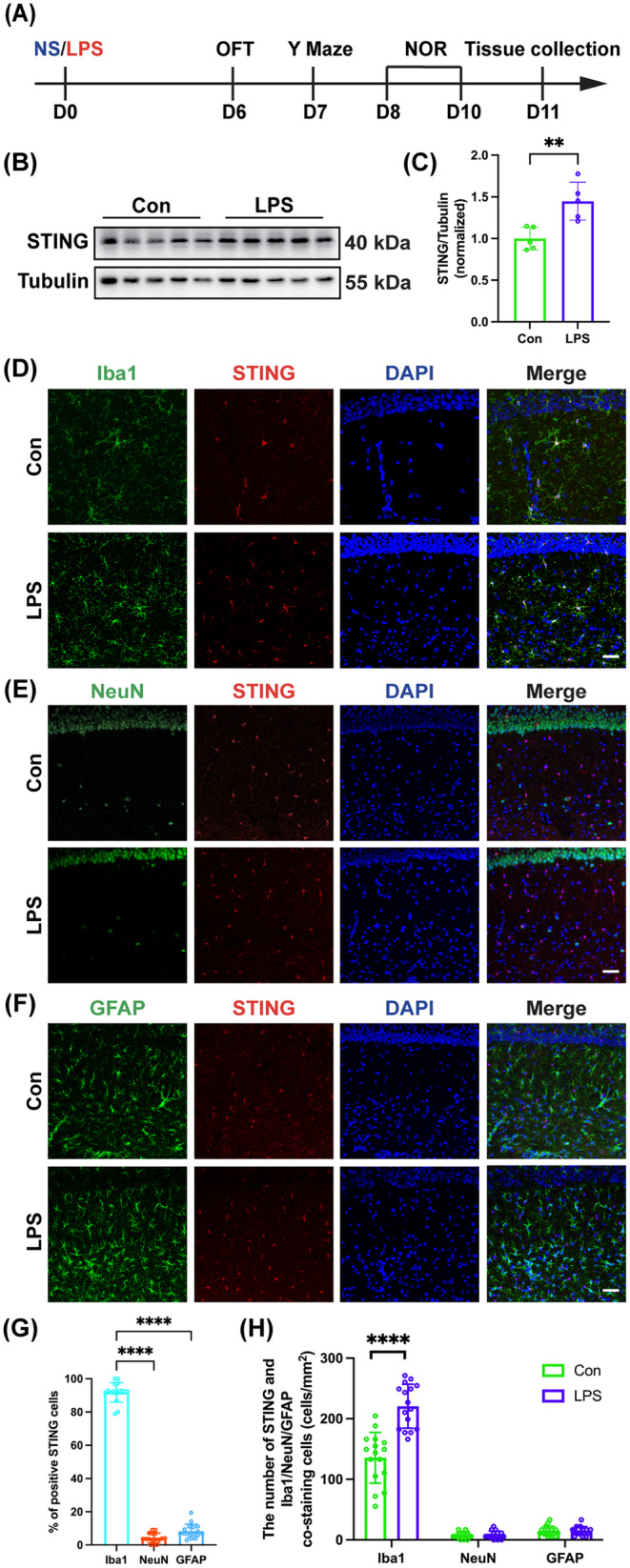
STING signaling was predominantly activated in microglia/macrophages in SAE mice. (A) Flowchart of the experiment 1. (B) Representative western blot bands of STING in the hippocampus. (C) Quantitative western blot analysis of STING expression in the hippocampus of the two groups. (D) Representative images of immunofluorescence staining of Iba1 (green), STING (red), DAPI (blue), and colocalization in the hippocampal CA1 region, scale bar = 50 μm. (E) Representative images of immunofluorescence staining of NeuN (green), STING (red), DAPI (blue), and colocalization in the hippocampal CA1 region, scale bar = 50 μm. (F) Representative images of immunofluorescence staining of GFAP (green), STING (red), DAPI (blue), and colocalization in the hippocampal CA1 region, scale bar = 50 μm. (G) Proportions of STING‐positive cells populations following LPS induction. (H) The number of STING and Iba1, NeuN, GFAP co‐staining cells in the hippocampal CA1 between the two groups. Data are shown as the mean ± SD (*n* = 4–5 mice/group). ****p < 0.0001 versus the indicated groups.

Following the experimental protocol (Figure 1A), it was found that the STING expression in the hippocampus was significantly increased in SAE mice (Figure 1B,C; *t* = 3.779, *p* < 0.01). To identify the cell type of STING expression in the hippocampus CA1 region, STING co‐labeling cell type‐specific markers NeuN (neurons), Iba1 (microglia/macrophages), or GFAP (astrocytes) were done and it was found that STING was predominantly expressed in the microglia/macrophages but very rare in neurons or astrocytes in the hippocampal CA1 region (Figure 1D–G; Iba1 vs. NeuN: *p* < 0.0001; Iba1 vs. GFAP: *p* < 0.0001). In comparison with the Con group, the number of STING colocalized with microglia/macrophages was significantly increased in the LPS group (Figure 1D,H; *t* = 6.146, *p* < 0.0001). However, the number of STING colocalized with neurons (Figure 1E,H; *t* = 0.5061, *p* = 0.6165) and the number of STING colocalized with astrocytes (Figure 1F,H; *t* = 0.08412, *p* = 0.9335) did not change significantly in the hippocampal CA1 after LPS injection. We also used TMEM119 to label microglia but TMEM119 neither specific nor reliable as a microglia marker under conditions of cellular stress according to the previous study [22], and found that TMEM119^+^ cells expressing STING accounted for the majority of Iba1^+^ cells expressing STING in both the Con group (Figure S1A,B; *t* = 18.38, *p* < 0.0001) and the LPS group (Figure S1C,D; *t* = 28.60, *p* < 0.0001). STING was shown to mainly locate in the endoplasmic reticulum [5]. To further pinpoint the subcellular localization of STING in microglia/macrophages and analyze the changes in the morphology of microglia/macrophages, Iba1, which is expressed in the cytoplasm, was selected instead of the cell surface protein TMEM119 [23].

### 3.2 STING Inhibitor C‐176 Treatment Attenuated LPS‐Induced Cognitive Impairment

**FIGURE 2:**
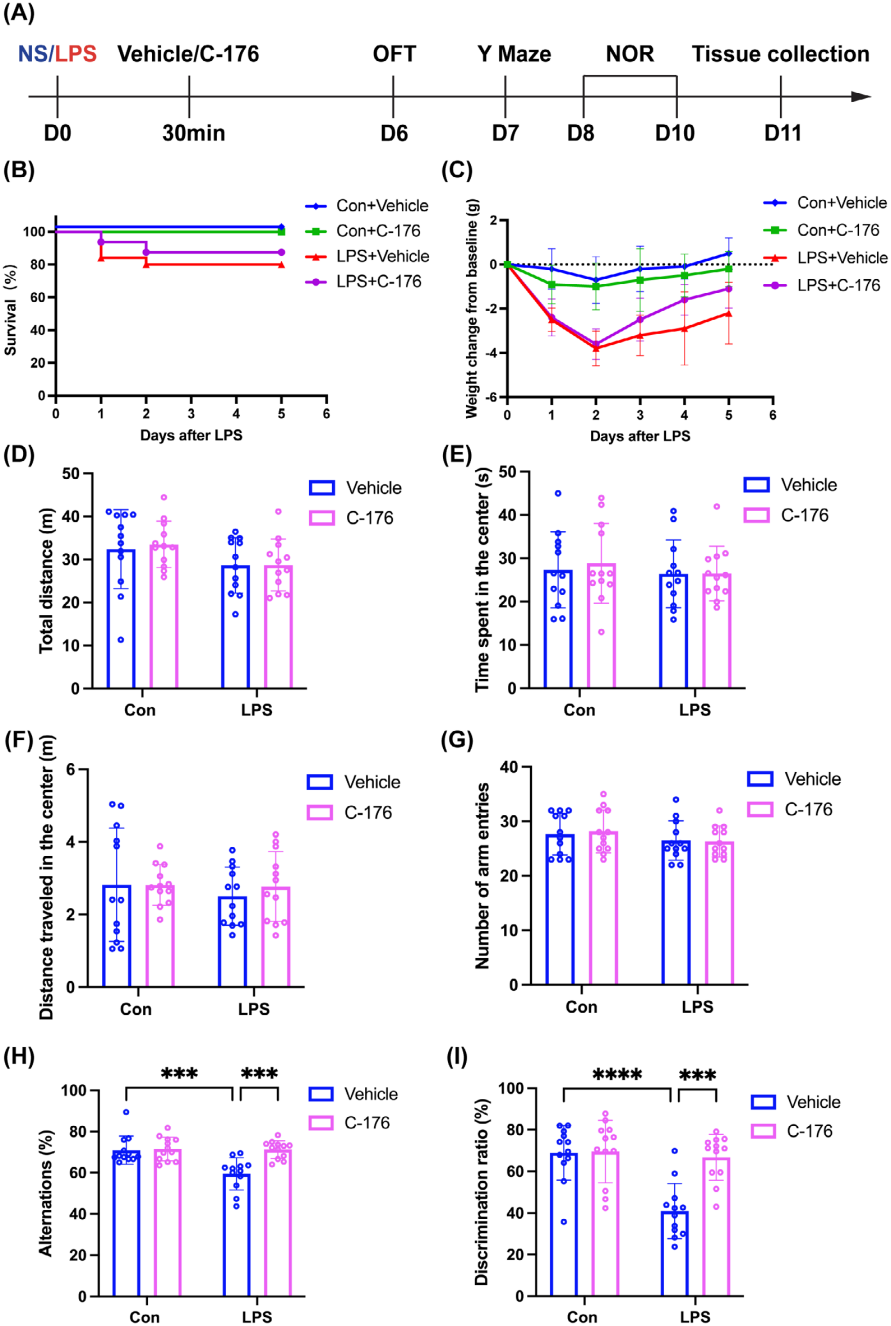
STING inhibitor C‐176 ameliorated LPS‐induced cognitive impairment. (A) Flowchart of the experiment 2. (B) Comparison of survival rate among the four groups. (C) Comparison of body weight changes among the four groups. (D–F) Performance of OFT among the four groups. (G and H) Performance of Y maze test among the four groups. (I) Performance of NOR test among the four groups. Data are expressed as mean ± SD (*n* = 10–12 mice/group). ***p < 0.001 and ****p < 0.0001 versus the indicated groups.

After 6 days of treatment following the experimental protocol (Figure 2A), no mice in the Con + Vehicle group or Con + C‐176 group died, whereas only 80% of mice in the LPS + Vehicle group survived. However, the administration of STING inhibitor C‐176 to the SAE mice improved their survival, and the survival rate was 90% for the LPS + C‐176 group (Figure 2B). Additionally, following LPS administration, the mice experienced significant weight loss, but the LPS + C‐176 group showed recovery from weight loss compared to the LPS + Vehicle group (Figure 2C). In the OFT test, there was no difference in total distance (Figure 2D; interaction: LPS × C‐176, *F*(1, 44) = 0.06674, *p* = 0.9793; LPS: *F*(1, 44) = 4.610, *p* = 0.0373; C‐176: *F*(1, 44) = 0.08573, *p* = 0.7710), time spent in the center (Figure 2E; interaction: LPS × C‐176, *F*(1, 44) = 0.09662, *p* = 0.7574; LPS: *F*(1, 44) = 0.4779, *p* = 0.4930; C‐176: *F*(1, 44) = 0.1210, *p* = 0.7296), or distance traveled in the center (Figure 2F; interaction: LPS × C‐176, *F*(1, 44) = 0.2049, *p* = 0.6530; LPS: *F*(1, 44) = 0.3617, *p* = 0.5506; C‐176: *F*(1, 44) = 0.1919, *p* = 0.6635) among the four groups, which excluded the possible effects of locomotor activity on behavioral results. In the Y maze test, there was no difference in the number of arm entries (Figure 2G; interaction: LPS × C‐176, *F*(1, 44) = 0.1040, *p* = 0.7486; LPS: *F*(1, 44) = 2.106, *p* = 0.1538; C‐176: *F*(1, 44) = 0.026, *p* = 0.8726). Furthermore, the LPS + Vehicle group exhibited a decrease of spontaneous alternation compared to the Con + Vehicle group, which was reversed with C‐176 treatment (Figure 2H; interaction: LPS × C‐176, *F*(1, 44) = 9.527, *p* = 0.0035; LPS: *F*(1, 44) = 10.44, *p* < 0.01; C‐176: *F*(1, 44) = 11.50, *p* < 0.0001). In the NOR test, mice in the LPS + Vehicle group showed a reduction in exploration time of novel object compared to the Con + Vehicle group, but this effect was reversed by C‐176 treatment (Figure 2I; interaction: LPS × C‐176, *F*(1, 44) = 11.10, *p* = 0.0018; LPS: *F*(1, 44) = 16.38, *p* < 0.001; C‐176: *F*(1, 44) = 12.05, *p* < 0.01). Taken together, these data indicated that STING played a significant role in cognitive impairment caused by LPS, which was ameliorated by C‐176 treatment.

### 3.3 STING Inhibitor C‐176 Ameliorated the Decreased Theta Oscillation in the Hippocampal CA1 Region of SAE Mice

**FIGURE 3:**
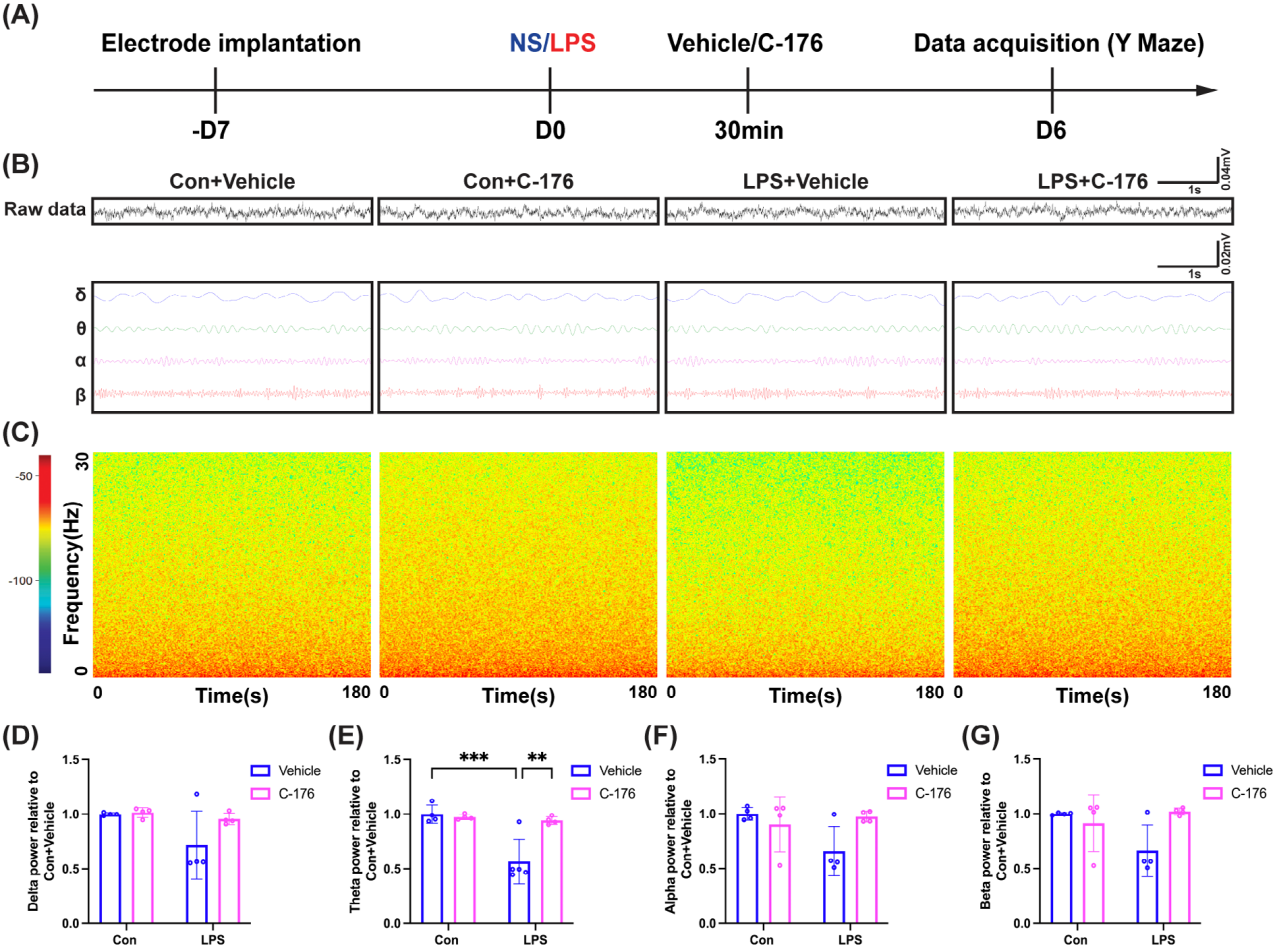
STING inhibitor C‐176 reversed abnormal theta oscillation in the hippocampal CA1 in SAE mice. (A) Flowchart of the electrophysiological experiment. (B) Representative images of LFP and filtered delta, theta, alpha, and beta oscillation in the hippocampal CA1 among the four groups. (C) Time‐frequency diagrams of LFP in the hippocampal CA1. (D–G) Quantification of average delta, theta, alpha, and beta oscillation powers in the hippocampal CA1 among the four groups. Data are expressed as mean ± SD (*n* = 4–5 mice/group). **p < 0.01 and ***p < 0.001 versus the indicated groups.

To further investigate whether working memory impairment in SAE mice was associated with changes in oscillatory power in the hippocampal CA1, local field potential (LFP) was tested while the mice performed the Y maze test (Figure 3A–C). We observed a significant decrease in theta oscillation power in the LPS + Vehicle group compared to the Con + Vehicle group. However, the administration of C‐176 effectively reversed this reduction (Figure 3E; interaction: LPS × C‐176, *F*(1, 13) = 11.47, *p* = 0.0049; LPS: *F*(1, 13) = 15.24, *p* < 0.05; C‐176: *F*(1, 13) = 8.657, *p* < 0.05). Moreover, there were no significant differences in delta, alpha, and beta oscillation powers among the four groups (Figure 3D; interaction: LPS × C‐176, *F*(1, 12) = 2.004, *p* = 0.1823; LPS: *F*(1, 12) = 4.536, *p* = 0.0546; C‐176: *F*(1, 12) = 2.537, *p* = 0.1372; Figure 3F; interaction: LPS × C‐176, *F*(1, 12) = 5.620, *p* = 0.0354; LPS: *F*(1, 12) = 2.415, *p* = 0.1461; C‐176: *F*(1, 12) = 1.600, *p* = 0.2299; Figure 3G; interaction: LPS × C‐176, *F*(1, 12) = 6.343, *p* = 0.0270; LPS: *F*(1, 12) = 1.700, *p* = 0.2167; C‐176: *F*(1, 12) = 2.395, *p* = 0.1476). These findings suggested that the cognitive impairment induced by LPS may be related to abnormal theta oscillation in the hippocampal CA1 region, and this alteration can be reversed by C‐176.

### 3.4 STING Inhibitor C‐176 Ameliorated STING Expression and Activation of Microglia/Macrophages in the Hippocampal CA1 Region of SAE Mice

**FIGURE 4:**
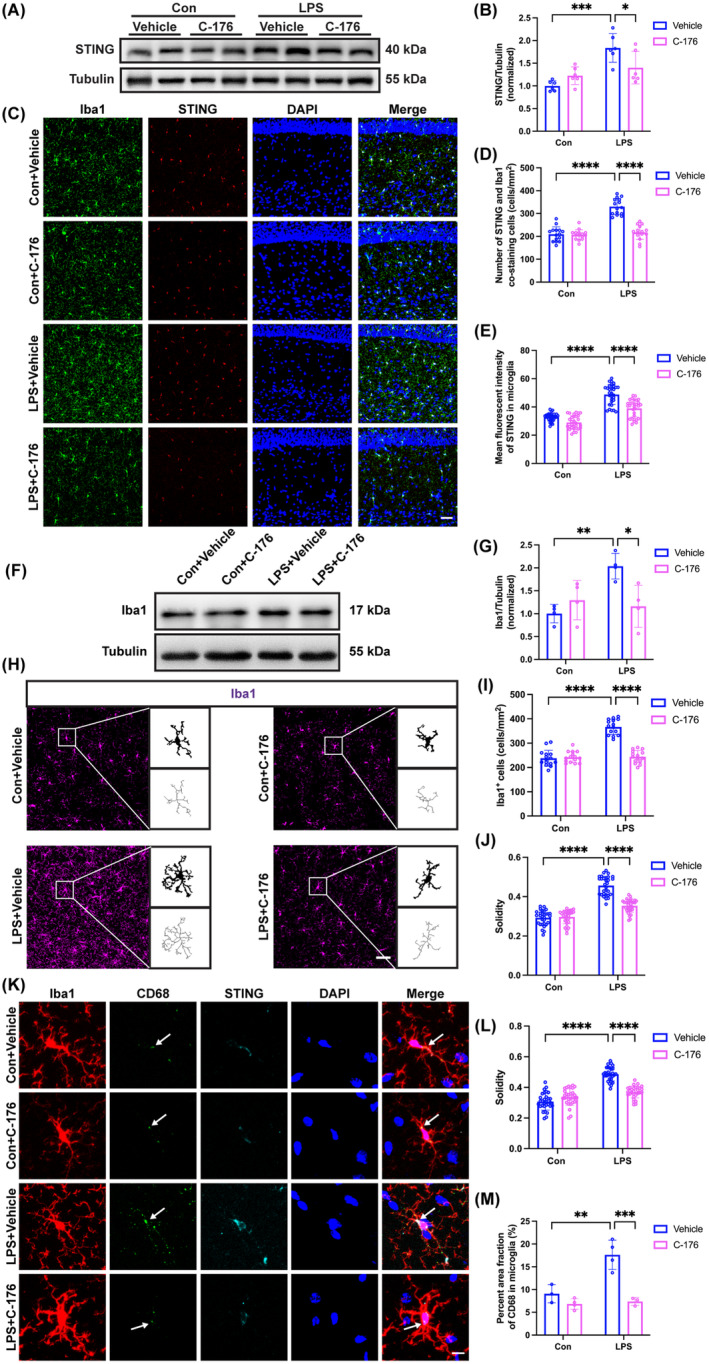
STING inhibitor C‐176 ameliorated STING overexpression and microglia/macrophages activation in SAE mice. (A) Representative western blot bands of STING in the hippocampus among the four groups. (B) Quantitative western blot analysis of STING expression in the hippocampus among the four groups. (C) Representative images of immunofluorescence staining of Iba1 (green), STING (red), DAPI (blue), and colocalization in the hippocampal CA1, scale bar = 50 μm. (D) Quantification of the number of STING and Iba1 co‐staining cells in the hippocampal CA1 among the four groups. (E) Quantification of the mean fluorescent intensity of STING in microglia/macrophages in the hippocampal CA1 among the four groups. (F) Representative western blot bands of Iba1 in the hippocampus among the four groups. (G) Quantitative western blot analysis of STING expression in the hippocampus among the four groups. (H) Representative images of immunofluorescence staining of Iba1 (magenta) and skeletonization of Iba1^+^ cells in the hippocampal CA1, scale bar = 50 μm. (I) Quantification of the number of Iba1^+^ cells in the hippocampal CA1 among the four groups. (J) Quantification of the solidity of Iba1^+^ cells in the hippocampal CA1 among the four groups. (K) Representative images of immunofluorescence staining of Iba1 (red), CD68 (green), STING (cyan), DAPI (blue), and colocalization in the hippocampal CA1, scale bar = 10 μm. (L) Quantification of the solidity of Iba1^+^ cells expressing STING in the hippocampal CA1 among the four groups. (M) Quantification of the percent area fraction of CD68 within microglia/macrophages in the hippocampal CA1 among the four groups. Data are expressed as mean ± SD (*n* = 3–6 mice/group). *p < 0.05, **p < 0.01, ***p < 0.001, and ****p < 0.0001 versus the indicated groups.

A significant increase in STING protein expression in the LPS + Vehicle group compared to the Con + Vehicle group. Whereas C‐176 treatment reduced the levels of STING protein (Figure 4A,B; interaction: LPS × C‐176, *F*(1, 20) = 9.448, *p* = 0.0060; LPS: *F*(1, 20) = 22.21, *p* < 0.001; C‐176: *F*(1, 20) = 0.9606, *p* = 0.3387). In addition, in comparison with the Con + Vehicle group, the number of STING and Iba1 co‐staining cells (Figure 4C,D; interaction: LPS × C‐176, *F*(1, 56) = 48.98, *p* < 0.0001; LPS: *F*(1, 56) = 69.75, *p* < 0.0001; C‐176: *F*(1, 56) = 53.58, *p* < 0.0001) as well as the mean fluorescent intensity of STING in microglia/macrophages (Figure 4C,E; interaction: LPS × C‐176, *F*(1, 116) = 9.315, *p* = 0.0028; LPS: *F*(1, 116) = 171.1, *p* < 0.0001; C‐176: *F*(1, 116) = 46.78, *p* < 0.0001) were significantly increased in the LPS + Vehicle group, which were reduced by C‐176 utilization. Western blot analysis indicated that in comparison with the Con + Vehicle group, the expression of Iba1 was significantly increased in the LPS + Vehicle group (Figure 4F,G; interaction: LPS × C‐176, *F*(1, 12) = 10.67, *p* = 0.0067; LPS: *F*(1, 12) = 6.342, *p* < 0.05; C‐176: *F*(1, 12) = 2.620, *p* = 0.1315). Consistent with these western blot results, we observed a remarkable increase in the number of microglia/macrophages (Figure 4H,I; interaction: LPS × C‐176, *F*(1, 56) = 75.60, *p* < 0.0001; LPS: *F*(1, 56) = 80.04, *p* < 0.0001; C‐176: *F*(1, 56) = 65.45, *p* < 0.0001) and the solidity of microglia/macrophages (Figure 4H,J; interaction: LPS × C‐176, *F*(1, 116) = 57.49, *p* < 0.0001; LPS: *F*(1, 116) = 242.2, *p* < 0.0001; C‐176: *F*(1, 116) = 47.72, *p* < 0.0001) in the LPS + Vehicle group relative to the Con + Vehicle group. Nevertheless, the C‐176 treatment restored these effects. Analogously, In the LPS + Vehicle group, the solidity of microglia/macrophages with increased STING expression was elevated in the hippocampal CA1 (Figure 4K,L; interaction: LPS × C‐176, *F*(1, 116) = 68.47, *p* < 0.0001; LPS: *F*(1, 116) = 141.8, *p* < 0.0001; C‐176: *F*(1, 116) = 24.22, *p* < 0.0001). By contrast, C‐176 treatment suppressed this effect. Furthermore, there was a notable increase in the levels of CD68, as represented by the area percentage of CD68 in Iba1^+^ cells expressing STING in the LPS + Vehicle group compared to the Con + Vehicle group. However, this situation was reversed by C‐176 treatment (Figure 4K,M; interaction: LPS × C‐176, *F*(1, 10) = 12.08, *p* = 0.0060; LPS: *F*(1, 10) = 15.70, *p* < 0.01; C‐176: *F*(1, 10) = 29.88, *p* < 0.001). These findings collectively supported that C‐176 reversed the elevated expression of STING in microglia/macrophages and activation of microglia/macrophages induced by LPS in the hippocampus.

### 3.5 STING Inhibitor C‐176 Attenuated Microglia/Macrophages‐Mediated Engulfment of Synaptic Structure in the Hippocampus of SAE Mice

**FIGURE 5:**
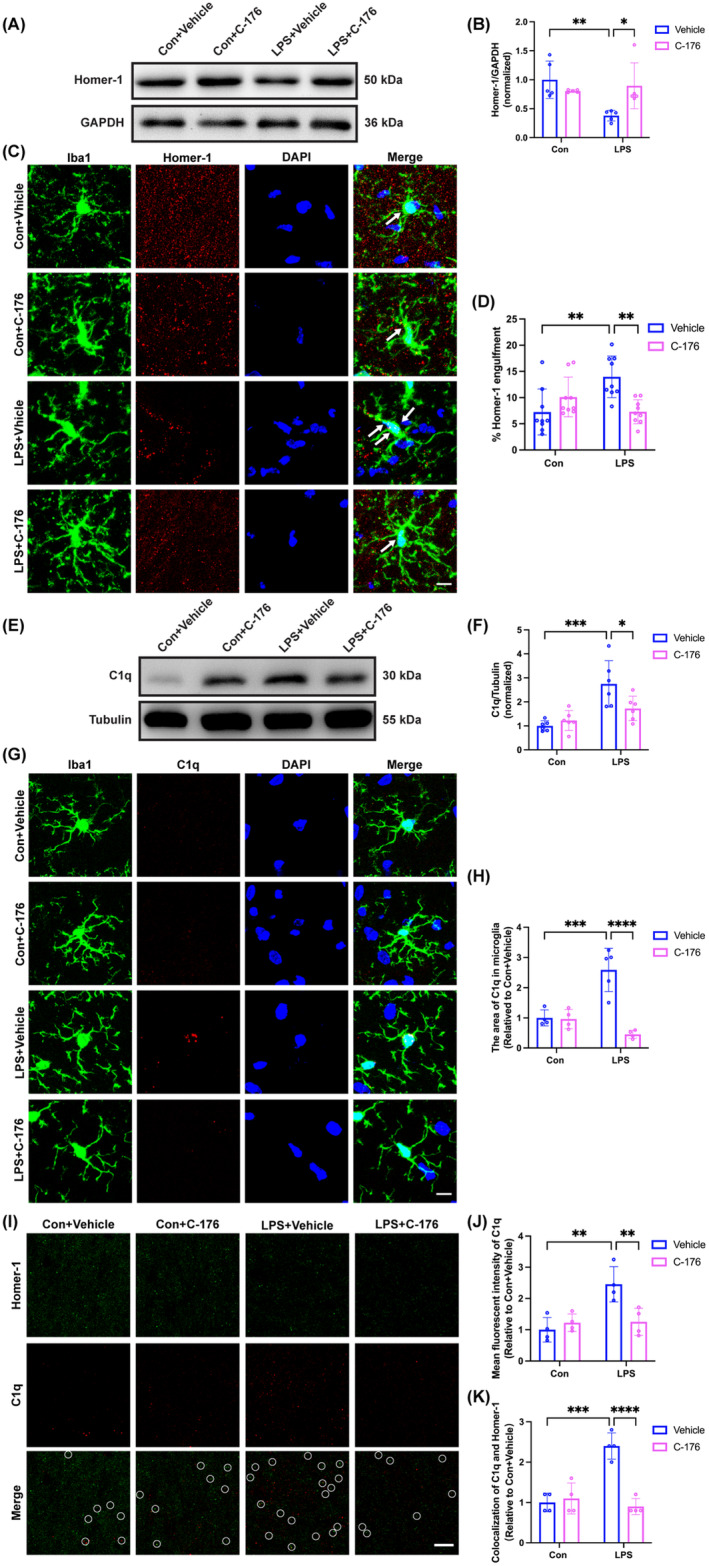
STING inhibitor C‐176 reduced microglia‐mediated engulfment of synapses in the hippocampal CA1 region of SAE mice. (A) Representative western blot bands of Homer‐1 in the hippocampus among the four groups. (B) Quantitative western blot analysis of Homer‐1 expression in the hippocampus among the four groups. (C) Representative images of immunofluorescence staining of Iba1 (green), Homer‐1 (red), DAPI (blue), and colocalization in the hippocampal CA1, scale bar = 10 μm. (D) Quantification of the percentage of Homer‐1 engulfment within Iba1^+^ cells in the hippocampal CA1 among the four groups. (E) Representative western blot bands of C1q in the hippocampus among the four groups. (F) Quantitative western blot analysis of C1q expression in the hippocampus among the four groups. (G) Representative images of immunofluorescence staining of Iba1 (green), C1q (red), DAPI (blue), and colocalization in the hippocampal CA1, scale bar = 10 μm. (H) Quantification of the area of C1q in microglia in the hippocampal CA1 among the four groups. (I) Representative images of immunofluorescence staining of Homer‐1 (green), C1q (red), and colocalization in the hippocampal CA1, scale bar = 10 μm. (J) Quantification of the mean fluorescent intensity of C1q in the hippocampal CA1 among the four groups. (K) Quantification of the colocalization analysis of C1q and Homer‐1 in the hippocampal CA1 among the four groups. Data are expressed as mean ± SD (*n* = 4–6 mice/group). *p < 0.05, **p < 0.01, ***p < 0.001, and ****p < 0.0001 versus the indicated groups.

A remarkable decrease in the protein expression of Homer‐1 in the LPS + Vehicle group relative to the Con + Vehicle group (Figure 5A,B; interaction: LPS × C‐176, *F*(1, 16) = 9.284, *p* = 0.0077; LPS: *F*(1, 16) = 5.196, *p* < 0.05; C‐176: *F*(1, 16) = 1.902, *p* = 0.1868). While, a significant increase in engulfment of Homer‐1 by microglia/macrophages was observed in the LPS + Vehicle group compared to the Con + Vehicle group (Figure 5C,D; interaction: LPS × C‐176, *F*(1, 32) = 14.93, *p* = 0.0005; LPS: *F*(1, 32) = 2.515, *p* = 0.1226; C‐176: *F*(1, 32) = 2.365, *p* = 0.1339). Compared to the Con + Vehicle group, the protein expression of C1q in the hippocampus strikingly increased in the LPS + Vehicle group (Figure 5E,F; interaction: LPS × C‐176, *F*(1, 20) = 6.585, *p* = 0.0184; LPS: *F*(1, 20) = 21.47, *p* < 0.001; C‐176: *F*(1, 20) = 2.665, *p* = 0.1182). Similarly, there was a notable increase in the area of C1q within microglia/macrophages in the hippocampal CA1 in the LPS + Vehicle group compared to the Con + Vehicle group (Figure 5G,H; interaction: LPS × C‐176, *F*(1, 13) = 22.90, *p* = 0.0004; LPS: *F*(1, 13) = 6.108, *p* < 0.05; C‐176: *F*(1, 13) = 24.52, *p* < 0.001). The mean fluorescent intensity of C1q (Figure 5I,J; interaction: LPS × C‐176, *F*(1, 12) = 11.02, *p* = 0.0061; LPS: *F*(1, 12) = 11.94, *p* < 0.05; C‐176: *F*(1, 12) = 5.209, *p* < 0.05) and the number of C1q co‐labeled with Homer‐1 puncta (Figure 5I,K; interaction: LPS × C‐176, *F*(1, 12) = 29.54, *p* = 0.0002; LPS: *F*(1, 12) = 16.62, *p* < 0.05; C‐176: *F*(1, 12) = 22.62, *p* < 0.001) were increased in the LPS+ Vehicle group compared with the Con + Vehicle group. In contrast, the C‐176 treatment effectively reversed these effects. In concert with the above results, we concluded that the excessive phagocytosis by microglia/macrophages may be mediated by the increased levels of complement C1q due to STING activation in SAE mice, which was ameliorated by C‐176 treatment.

### 3.6 STING Inhibitor C‐176 Reversed LPS‐Induced Disruption of Synaptic Plasticity in the Hippocampus

**FIGURE 6:**
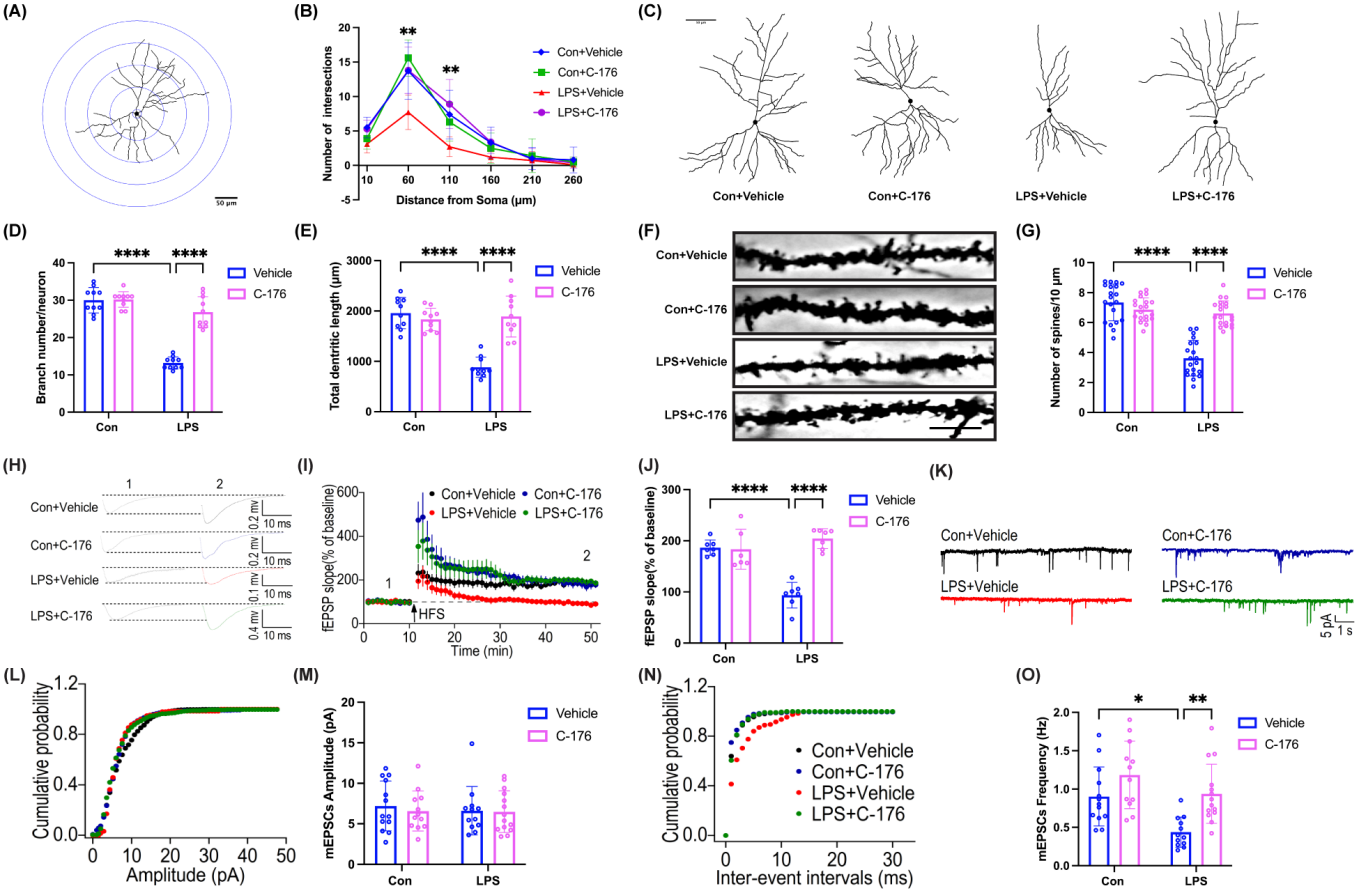
STING inhibitor C‐176 ameliorated disruption of synaptic plasticity in the hippocampal CA1 of SAE mice. (A) Sholl analysis pattern map of neuronal morphology, scale bar = 50 μm. (B) Quantification of dendritic intersections of neuronal dendrites in the hippocampal CA1 among the four groups. (C) Representative images of neuronal tracings in hippocampal CA1 region, scale bar = 50 μm. (D) Quantification of the number of neuronal branches in the hippocampal CA1 among the four groups. (E) Quantification of the total length of neuronal branches in the hippocampal CA1 among the four groups. (F) Representative images of the dendritic spines of hippocampal CA1 neurons, scale bar = 10 μm. (G) Quantification of dendritic spine density of neurons in the hippocampal CA1 among the four groups. (H) Schematic representation of field excitatory postsynaptic potentials (fEPSPs) before (1) and after (2) HFS among the four groups. (I) Time‐dependent changes in the slope of fEPSPs before (1) and after (2) HFS in hippocampal slices among the four groups. (J) Quantification of fEPSPs slopes evoked by HFS among the four groups. (K) Sample traces of mEPSCs were recorded from pyramidal neurons in the hippocampal CA1. (L and M) Quantification of mean amplitude of mEPSCs among the four groups. (N and O) Quantification of mean frequency of mEPSCs frequency among the four groups. Data are presented as the mean ± SD (*n* = 3–5 mice/group). *p < 0.05, **p < 0.01, and ****p < 0.0001 versus the indicated groups.

In the LPS + Vehicle group, there were visible decreases in the number of intersections at both 50 and 100 μm from the cell body compared to the Con + Vehicle group, but it was markedly increased by C‐176 treatment (Figure 6A,B; 50 μm: interaction: LPS × C‐176, *F*(1, 36) = 4.265, *p* = 0.0462; LPS: *F*(1, 36) = 14.71, *p* < 0.001; C‐176: *F*(1, 36) = 15.48, *p* < 0.001; 100 μm: interaction: LPS × C‐176, *F*(1, 36) = 15.20, *p* = 0.0004; LPS: *F*(1, 36) = 1.258, *p* = 0.2695; C‐176: *F*(1, 36) = 7.420, *p* < 0.01). Compared with the Con + Vehicle group, dramatic decreases in the number of neuronal branches (Figure 6C,D; interaction: LPS × C‐176, *F*(1, 36) = 49.75, *p* < 0.0001; LPS: *F*(1, 36) = 113.1, *p* < 0.0001; C‐176: *F*(1, 36) = 52.77, *p* < 0.0001), the total dendritic length (Figure 6C,E; interaction: LPS × C‐176, *F*(1, 36) = 36.56, *p* < 0.0001; LPS: *F*(1, 36) = 29.49, *p* < 0.0001; C‐176: *F*(1, 36) = 22.04, *p* < 0.0001), and the dendritic spine density of pyramidal neurons (Figure 6F,G; interaction: LPS × C‐176, *F*(1, 76) = 56.84, *p* < 0.0001; LPS: *F*(1, 76) = 74.89, *p* < 0.0001; C‐176: *F*(1, 76) = 30.00, *p* < 0.0001) were observed in the LPS + Vehicle group, which increased significantly by C‐176 treatment. In addition, the LPS + Vehicle group showed significantly weaker LTP compared to the Con + Vehicle group after LPS administration. However, the presence of C‐176 attenuated the extent of LPS‐induced LTP impairment (Figure 6H–J; interaction: LPS × C‐176, *F*(1, 23) = 33.44, *p* < 0.0001; LPS: *F*(1, 23) = 13.39, *p* < 0.05; C‐176: *F*(1, 23) = 29.79, *p* < 0.0001). The mean amplitude of mEPSCs did not differ significantly among the four groups (Figure 6K–M; interaction: LPS × C‐176, *F*(1, 47) = 0.1050, *p* = 0.7474; LPS: *F*(1, 47) = 0.1867, *p* = 0.6676; C‐176: *F*(1, 47) = 0.2337, *p* = 0.6311). However, the mean frequency of mEPSCs was significantly reduced in the LPS + Vehicle group compared to the Con + Vehicle group, and this effect was reversed by C‐176 (Figure 6N,O; interaction: LPS × C‐176, *F*(1, 47) = 1.138, *p* = 0.2916; LPS: *F*(1, 47) = 12.07, *p* < 0.01; C‐176: *F*(1, 47) = 14.54, *p* < 0.001). Overall, these results indicated that STING contributed to synaptic dysfunction and impaired excitatory synaptic transmission in SAE mice, which can be alleviated by C‐176.

## 4 Discussion

In the present study, we uncovered the role of STING in microglia/macrophages associated with cognitive impairment in SAE mice. Our results demonstrated that overexpression of STING in microglia/macrophages mediated their activation and then secreted complement C1q, which led to phagocytosis of excitatory synapses, resulting in neural network abnormalities, and ultimately contributed to the development of SAE. Our work further demonstrated that blockage of STING with a specific inhibitor C‐176 significantly reversed these changes and improved cognitive function in SAE mice.

The new definition of sepsis emphasizes the importance of an aberrant immune response to infection, which leads to life‐threatening organ dysfunction and enhances the pathogenesis and progression of sepsis including SAE [24]. SAE is associated with increased mortality, extensive hospital cost, and prolonged hospitalization, followed by persistent cognitive impairment and limitations in physical function [25]. In our study, mice injected with LPS developed cognitive impairment as assessed with Y maze and NOR test. Our findings are consistent with previous studies demonstrating that LPS‐induced sepsis can lead to cognitive impairment [26]. However, the precise molecular mechanisms remain unclear.

The hippocampus, being a component of the brain's limbic system, is accountable for the bulk of learning and memory procedures [27]. Specifically, the CA1 region performs a crucial role in the establishment of long‐term memories [28]. Neuronal activity in the cerebral cortex demonstrates rhythms or oscillations that mirror periodic variations in the excitability of neuronal populations [29]. These neural rhythms are thought to underlie perceptual and cognitive processes in both animals and humans [30, 31]. Theta oscillation (4–7 Hz), a prominent rhythm in the hippocampus, has been extensively investigated during working memory processes in humans [32]. Theta oscillation was enhanced in response to demanding task challenges [33], and greater theta power elicited during encoding indicated successful memory retrieval [32]. In our study, we found significantly decreased theta oscillation during locomotion in LPS‐challenged mice but the delta, alpha, and beta oscillation powers were not significantly affected. Notably, we found that abnormal theta oscillation was closely related to cognitive impairment in SAE mice. Additionally, it was found that a decrease in theta oscillation was also observed in the cecal ligation and puncture model [20] and in the model with LPS injection [34]. Nevertheless, the mechanism responsible for the abnormal theta oscillation remains to be explored.

Synapses are the pivotal sites for functional connections and information transmission among neurons. The majority of excitatory synapses in the mammalian CNS are located on dendritic spines, making spines convenient indicators of excitatory synaptic presence [35]. Synaptic dysfunction is closely linked to cognitive impairment [36]. During synaptic pruning, microglia/macrophages play a role in phagocytosing synaptic material, which is essential for the proper development and maintenance of brain circuitry [37]. Studies have demonstrated that activated microglia/macrophages disrupted synaptic plasticity and decreased the number of dendritic spines, ultimately resulting in cognitive impairment [38]. In the present study, excitatory synapses were engulfed by microglia/macrophages following LPS. However, we cannot rule out the effect of activated microglia/macrophages on inhibitory synapses, as well as the efficiency of excitatory synaptic transmission also decreased. Microglia/macrophages play a crucial role in shaping and developing synaptic circuits, a process vital for establishing precise synaptic connectivity. Recent research suggested a potential involvement of the complement cascade in this process [39]. In addition to the physiological conditions, positive signals that modulate microglia/macrophages‐mediated synaptic elimination also play critical roles in some disease‐related neuropsychiatric symptoms. For instance, an abnormal increase of the C1q level in neuronal synapses leads to early synaptic loss mediated by microglia/macrophages [16]. Among the innate immune molecules implicated is complement cascade component C1q, which is commonly referred to the “eat me” signal because it promotes phagocytosis by binding to unwanted or harmful material [40]. In our study, we discovered that complement C1q mediated synaptic phagocytosis by microglia/macrophages. Although microglia/macrophages are considered to be the main source of C1q in the brain [41], we cannot exclude the influence of other cells on C1q expression. Nevertheless, the mechanism that mediates the overactivation of microglia/macrophages leading to complement‐mediated synaptic phagocytosis remains to be further explored.

STING, an endoplasmic reticulum adaptor protein, acts as a central immune molecule, usually in a state of self‐inhibition, and has been predominantly found in the endoplasmic reticulum [42]. Upon binding to cGAS‐catalyzed second messenger 2′3′‐cGAMP, STING promptly triggers the inflammatory cascade through NF‐κB signaling or other pathways [43, 44]. STING has thus emerged as an attractive target for drug discovery, particularly in cancer therapy [45]. Nevertheless, scant information is available regarding the role of STING in the brain, and whether it contributes to neuroinflammation and neurodegeneration remains unknown. In our experiment, immunofluorescence analysis revealed that STING was primarily colocalized with microglia/macrophages in the hippocampus, which was in line with the most recently published study [46]. Therefore, we focused primarily on the role of STING in microglia/macrophages. In addition, recent studies indicated that STING activation promoted microglia/macrophages activation in diverse inflammatory models [10, 47], which supported our findings. C‐176, a covalent inhibitor of STING, can effectively inhibit the activation of STING [48]. Our study suggested that C‐176 might improve the cognitive impairment of the SAE mice by suppressing the activation of STING in microglia/macrophages in the CA1 region of the hippocampus, reducing excessive phagocytosis of synapses by microglia/macrophages and improving synaptic plasticity.

Our experimental data are decent in supporting our conclusions. However, overexpression of STING in wild‐type mice is helpful to understand whether STING plays a causal role in microglia/macrophages activation and subsequent cognitive impairment. Another limitation is that our model with LPS injection cannot mimic clinical settings. All these require exploration in our future studies.

In summary, our work demonstrated that overexpression of STING led to microglia/macrophages activation, triggering the complement C1q cascade, and resulting in synaptic loss as well as aberrant theta oscillation, ultimately causing cognitive impairment in SAE mice. Importantly, the administration of STING inhibitor C‐176 effectively alleviated the pathological changes and cognitive impairment induced by sepsis. This study provides novel insights into the role of STING regulation in microglia/macrophages synapse elimination and provides new findings of STING in the SAE. The data may be helpful to further our understanding of cognitive impairment associated with sepsis.

## Author Contributions

Xin Lv and Min Jia designed this work. Xin Lv, Min Jia, and Xiao Feng performed the experiments. Xin Lv, Jia‐xiong Jian, and Jian‐jun Yang analyzed the data. Xin Lv, Mu‐huo Ji, and Da‐qing Ma wrote the paper. Jin‐chun Shen, Yu‐gang Diao, and Mu‐huo Ji supervised the overall experiment. All authors read and approved the final manuscript.

## Conflicts of Interest

The authors declare no conflicts of interest.

## Supporting information


**Figure S1.** TMEM119^+^ cells expressing STING accounted for the majority of Iba1^+^ cells expressing STING.

## Data Availability

Data supporting the findings of this study are available within the article.
