# Supplementary material for: STING Driving Synaptic Phagocytosis of Hippocampal Microglia/Macrophages Contributes to Cognitive Impairment in Sepsis‐Associated Encephalopathy in Mice
Source: CNS Neurosci Ther. 2024 Dec 19;30(12):e70166. doi: 10.1111/cns.70166 (PMC11656402; doi:10.1111/cns.70166)
Supplement: Supplementary file 1 — Figure S1. TMEM119+ cells expressing STING accounted for the majority of Iba1+ cells expressing STING. [file CNS-30-e70166-s001.docx]

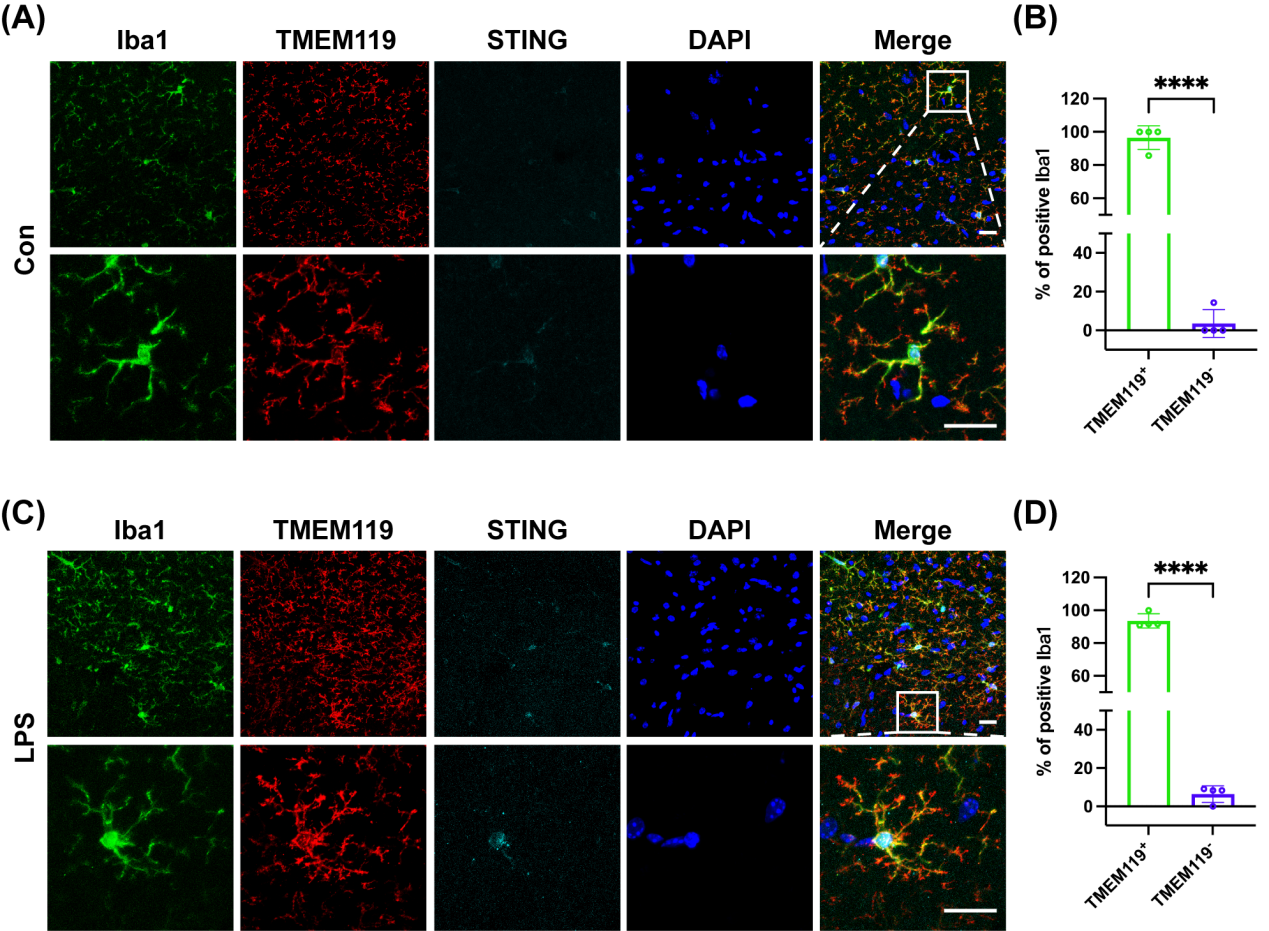


**Figure S1.** TMEM119^+^ cells expressing STING accounted for the majority of Iba1^+^ cells expressing STING. (A) Representative images of immunofluorescence staining of Iba1 (green), TMEM119 (red), STING (cyan), DAPI (blue) and colocalization in the hippocampal CA1 region in Con group, scale bar = 20 μm. (B) Quantification of the proportion of TMEM119^+^ cells and TMEM119^-^ cells in Iba1^+^ cells in the Con group. (C) Representative images of immunofluorescence staining of Iba1 (green), TMEM119 (red), STING (cyan), DAPI (blue) and colocalization in the hippocampal CA1 region in LPS group, scale bar = 20 μm. (D) Quantification of the proportion of TMEM119^+^ cells and TMEM119^-^ cells in Iba1^+^ cells in the LPS group. Data are shown as the mean ± SD (n = 4 mice/group). *****P* < 0.0001 vs. the indicated groups
